# Plateau pikas lead to higher rhizome and root-derived bud densities and their contribution to total belowground bud density in alpine grasslands

**DOI:** 10.3389/fpls.2025.1567822

**Published:** 2025-05-20

**Authors:** Ni Wang, Qian Wang, Xiao Pan Pang, Zheng Gang Guo

**Affiliations:** ^1^ Engineering Research Center of Grassland Industry, Ministry of Education, College of Pastoral Agriculture Science and Technology, Lanzhou University, Lanzhou, China; ^2^ State Key Laboratory of Herbage Improvement and Grassland Agro-ecosystems, College of Pastoral Agriculture Science and Technology, Lanzhou University, Lanzhou, China

**Keywords:** tiller bud, rhizome bud, root-derived bud, total belowground bud, small semi-fossorial herbivores

## Abstract

Plant belowground buds are important agents for examining the effects of small semi-fossorial herbivores on plant population regeneration in perennial grasslands. In this study, we used the plateau pika (*Ochotona curzoniae*) as a focally small semi-fossorial herbivore to investigate its effects on tiller, rhizome, and root-derived bud densities. The study was conducted in alpine grasslands from Gangcha County to Gonghe County in Qinghai Province, China. A paired experimental design was employed, with 20 plots established in areas with plateau pikas and 20 plots plateau pikas established in without plateau pikas. The results indicated that total belowground, rhizome, and root-derived bud densities were 49.31%, 84.68%, and 91.73% higher in the presence than in absence of plateau pikas, respectively. The contributions of rhizome and root-derived buds to total belowground buds were 8% and 3% higher in the presence than in the absence of plateau pikas, respectively, whereas the contribution of tiller buds to total belowground buds was 11% lower in the presence than in the absence of plateau pikas. Total belowground buds were positively correlated with soil moisture and negatively correlated with plant aboveground biomass in the absence of plateau pikas. In contrast, they were positively correlated with total soil phosphorus and rhizome biomass in the presence of plateau pikas. These results suggested that the phosphorus supplementation is an alternative way to improve plant population regeneration and facilitate alpine grassland sustainability when plateau pikas are present in alpine grasslands.

## 1 Introduction

Herbivores have important impacts on plant population regeneration and plant community structure (Davidson et al., 2012; Case et al., 2013; Niu et al., 2019), which frequently contribute to the long-term sustainability of perennial grasslands (Benson et al., 2004). Belowground buds are vital for explaining how herbivores affect plant population regeneration and plant community maintenance in perennial grasslands (Wang et al., 2018). Based on the position at which the buds are borne in perennial grasslands (Wang et al., 2018; Ma et al., 2023), belowground plant buds are usually categorized into tiller, rhizome, root-derived, and bulb buds (Wang et al., 2018; Qian et al., 2017), although belowground plant buds can be sorted into various types with different taxonomic categories (Ott et al., 2019). Rhizome and root-derived buds facilitate plant population regeneration and expansion because plant rhizomes often expand their spatial scope in soils to find newly available resources (Ma et al., 2019). In contrast, tiller and bulb buds contribute to the persistence of individual populations by producing phalanx tillers (Ott and Hartnett, 2015) and new bulbs (Ma et al., 2023).

Large herbivore grazing affects total belowground bud density (Dalgleish and Hartnett, 2006, 2009). Previous studies have shown that simulation bison or cattle grazing leads to grass bud number per shoot does not affect sedge and forb bud number per shoot within the tallgrass prairie region of northeastern Kansas, USA (VanderWeide and Hartnett, 2015), whereas mixture grazing of sheep and cattle leads to a decrease in tiller, rhizome, root-derived, and bulb bud densities in a steppe on China’s Loess Plateau (Zhao et al., 2017).

Countless small semi-fossorial herbivores naturally live in vast grasslands throughout the world. These small herbivores have been verified to affect some plant population and plant community structures in perennial grassland (Davidson et al., 2012; Smith et al., 2019) although selectively consuming some plants (Yu et al., 2017; Moorhead et al., 1988), cutting tall-statured plants to find a predator (Pech et al., 2007), altering soil properties (Pang et al., 2020; Duan et al., 2024), producing fertile island (Galiano et al., 2014) and bare patches (Zhang et al., 2021), and constructing burrowing system (Wei and Zhang, 2018). The presence of small semi-fossorial herbivores increases the total belowground bud density of graminoids but does not influence that of forbs in alpine meadows (Wang et al., 2018). Total belowground bud density in perennial grasslands depends on a combination of changes in root-derived, tiller, rhizome, and bulb buds. However, how the presence of small semi-fossorial herbivores influences different types of belowground bud densities and their contribution to total belowground bud density in perennial grasslands is not well documented.

The total belowground bud density is related with soil water moisture (Dalgleish and Hartnett, 2006), nitrogen supply (Qian et al., 2021), soil carbon content (Li et al., 2021), soil organic carbon, and plant species richness (Wu et al., 2020). Furthermore, soil moisture content has been reported to correlate with rhizome and root-derived bud densities (Wu et al., 2021; Ma et al., 2023), whereas it correlates with tiller bud density in alpine meadows (Ma et al., 2023). Consequently, more studies are needed to simultaneously investigate the impact of small semi-fossorial herbivores on different types of belowground bud density and their contribution to the total belowground bud density. Additionally, research should test whether the presence of these herbivores influences the relationship between environmental factors and different types of belowground bud density. This will provide precise information for improving plant population regeneration and maintaining plant community structure when small semi-fossorial herbivores are present, facilitating perennial grassland sustainability.

Plateau pika (*Ochotona curzoniae*) is a semi-fossorial herbivore endemic to the Qinghai–Tibetan Plateau (Smith and Foggin, 1999) and is native to alpine grasslands, weighing approximately 150 g on average (Pech et al., 2007). This animal prefers to consume certain plants (Pang and Guo, 2017), clips tall-statured plants (Zhang et al., 2020), damages plant roots and rhizomes in the process of constructing burrow systems (Wang et al., 2018), and alters nutrient concentrations (Yu et al., 2017), which possibly impact on rhizome, tiller, root-derived, and bulb bud densities and their combinations.

In this study, the plateau pika was used as a focal species to explore the effect of the presence of a semi-fossorial herbivore on tiller, rhizome, root-derived, and bulb bud densities, and their combinations. We hypothesized that (1) the presence of plateau pikas leads to lower tiller, rhizome, root-derived, and bulb bud densities because mixture grazing of sheep and cattle decreases them (Zhao et al., 2017); (2) the presence of plateau pika does not change the contribution of tiller, rhizome, root-derived, and bulb bud densities to total belowground bud density because the four type buds simultaneously decrease; and (3) the presence of plateau pikas alters the relationship between environmental factors and total belowground bud density because the plateau pikas create an extensive disturbance on alpine grasslands (Wang et al., 2018).

## 2 Materials and methods

### 2.1 Study site description

**Figure 1:**
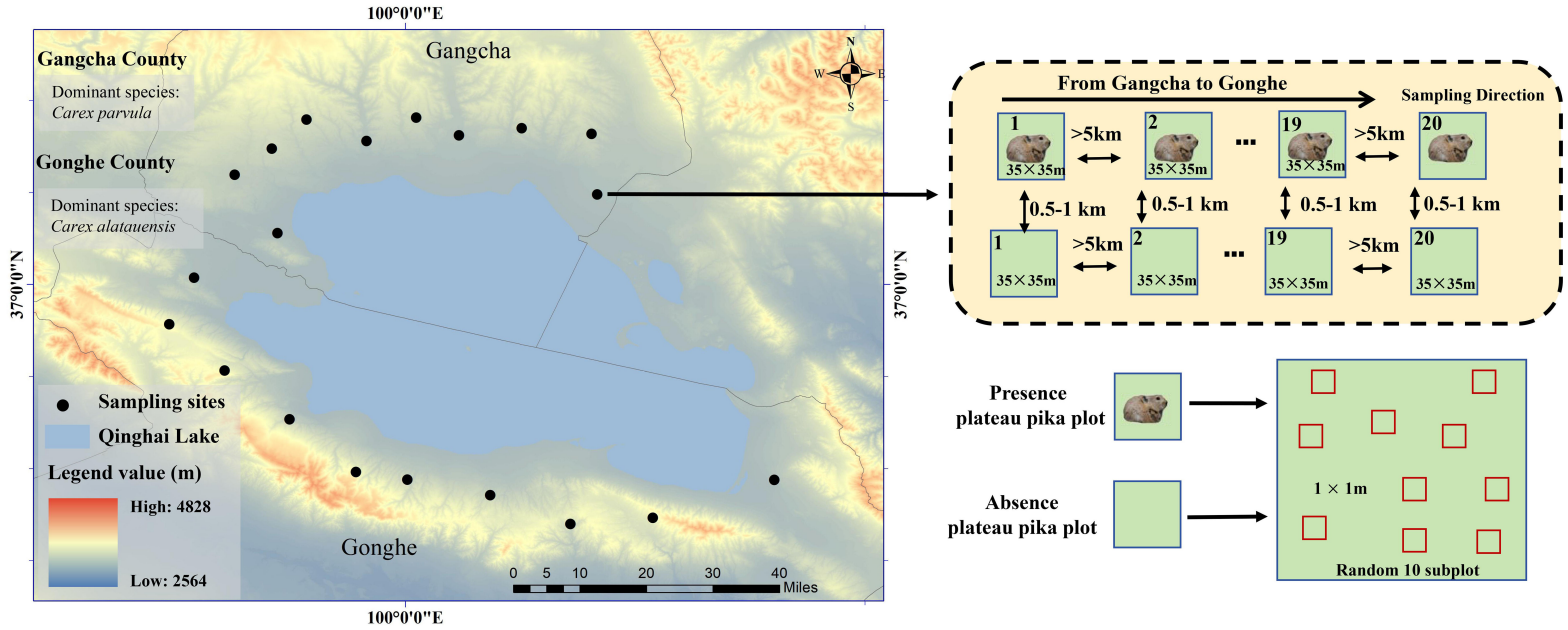
Sampling sites in the study area and sampling paired plots present and absent plateau pika.

Plateau pikas are often found in alpine grasslands with different soil types, topographies, and microclimates. Based on the presence of plateau pikas in previous studies (Wang et al., 2018), we selected Gangcha County and Gonghe County in the Qinghai Province of China to conduct field surveys and plot placement. The climate is typical of a continental plateau, and the elevation of the field survey area ranges from 3,265 to 3,750 m. The average annual temperature and annual mean precipitation is 1.0°C and 400 mm, respectively, in Gangcha County and 5.6°C and 502 mm in Gonghe County (**Figure 1**). Like Cambisols in the WRB classification, the soil type is categorized as alpine meadow soil under the Chinese Soil Classification System (Sorokin et al., 2021).

Most alpine grasslands in the two counties are dominated by tussock-forming *Carex parvula* in Gangcha County and rhizomatous *Carex alatauensis* in Gonghe County (**Figure 1**). Alpine grasslands are typically divided into cold- and warm-season grazing zones. Cold-season grazing areas are fenced from mid-April to early October, then opened for Tibetan sheep and yak grazing from late October to early April. Although several small herbivores are usually found in Gangcha County and Gonghe County, only plateau pikas were found in the field survey areas.

### 2.2 Experimental design

Plateau pikas live in families with approximately two to five adults (Pech et al., 2007; Zhou et al., 2023) and their offspring because young offspring do not disperse during the year of birth (Zhang et al., 2020; Pang et al., 2020). In addition, this animal prefers open and short vegetation habitats because they can easily spot predators and communicate with each other. Thus, plateau pikas are usually found in a territorial and patchy manner on alpine grasslands, and alpine grasslands without plateau pikas are easily found because the dispersal of plateau pikas is a gradually process.

This study used a randomly stratified and paired design to place the plots in cold-season grazing areas where no grazing occurred during the plant growth season. The first plot with plateau pikas, determined using visible plateau pikas or active burrow entrances, was randomly selected from the field survey areas of Gangcha County, with a size of 35 m × 35 m, comparable to the average home range of plateau pikas (Paruchuri et al., 2019; Duan et al., 2024). Next, a paired plot in the presence of plateau pikas was selected and identified by invisible plateau pikas or burrow entrances. The distance between the plot inhabited by plateau pikas and the corresponding paired plot without plateau pikas varied from 500 to 1,000 m. However, if the distance separating these two plots was too short, there was a possibility that plateau pikas could move freely between the plots, potentially affecting the experimental results by introducing unintended interactions or overlaps (Pang et al., 2020); if this distance was too large, vegetation characteristics and topography might be different between plots with the presence of plateau pikas and its paired plot. We attempted to find the second pair of plots along the road from Gangcha County to Gonghe County (**Figure 1**). The distance between the first and second paired plots exceeded 5 km, ensuring adequate spatial separation to reduce potential interactions or cross-influences. Similarly, a total of 40 paired plots were selected, consisting of 20 plots with plateau pikas present and 20 plots without them, providing a balanced and robust framework for comparative analysis in the study.

A total of 10 subplots, each measuring 1 m × 1 m, were randomly established within each experimental plot, both in areas inhabited by plateau pikas and in areas without their presence, to facilitate comparative analysis. If a subplot was placed on the burrow entrances, it was moved slightly to avoid the burrow entrance. Thus, 20 plots with plateau pikas contained different population densities of plateau pikas across various soil types, topographies, and microclimates, comprising 200 subplots each in the presence and absence of plateau pikas, which permitted a general pattern of the presence of plateau pikas in relation to different types of belowground buds in alpine grasslands on a large scale.

### 2.3 Field sampling

Plateau pikas cause the most disturbance in alpine grasslands and have the largest population density in August (Pang et al., 2020; Li et al., 2021; Zhou et al., 2023; Duan et al., 2024). Thus, field surveys and sampling took place in early August 2020.

First, active burrow entrances were recorded in each plot in the presence of plateau pikas using the three-day plugging burrows (Yu et al., 2017), and the active burrow entrance densities ranged from 550 to 650 per ha (Wang et al., 2018). In areas where plateau pikas are present, the survey showed that the densities of active burrow entrances in 20 plots were 620, 640, 559, 552, 641, 580, 635, 568, 594, 596, 648, 553, 585, 570, 600, 645, 590, 585, 627, and 615 per ha, from Gangcha County to Gonghe County. Second, 40 plots (20 plots with plateau pikas and 20 plots without plateau pikas) were established, each containing 10 subplots. In each subplot, a soil block (20 cm width × 20 cm length × 10 cm depth) was randomly dug at each subplot using a small shovel, packed into sealed cloth bags, and transported back to the lab in a portable refrigerator at 4°C. It was used to measure the belowground bud number of each bud type. A total of 10 soil blocks were collected per plot. All plant species recorded in the soil blocks and their taxonomic information was referenced from the Plants of the World online (https://powo.science.kew.org/) (**Supplementary Table S1**). Third, outside the soil block area in the same subplot, a 30 cm × 30 cm quadrat was used to harvest all aboveground plants for measuring aboveground biomass. Samples were placed in cloth bags and taken to the laboratory. A total of 10 aboveground biomass samples were collected per plot. Fourth, in the same quadrat, a root auger with an inner diameter of 10 cm was used to randomly collect soil columns from the 0–10-cm soil layer in each subplot. Each soil column was placed in a cloth bag and transported to the laboratory for determining belowground plant biomass. A total of 10 soil columns were collected per plot. Fifth, a stainless-steel cutting ring (100 cm^3^ in volume) was used to collect soil samples from the soil profile created by the root auger. These samples were stored in sealed plastic bags for measuring soil bulk density and soil water content. A total of 10 samples per plot were collected for these measurements. Finally, a soil auger with a 5-cm diameter was used to collect five replicate soil samples from the 0–10-cm layer in each subplot. The five samples were mixed to form a composite sample, which was stored in sealed plastic bags and used to measure soil physical and chemical properties. A total of 10 composite samples were collected per plot.

### 2.4 Sample analyses

All samples were processed within 2 weeks in the laboratory. The soil block was carefully cleaned with tap water to separate the belowground plant organs and pruned roots from the soil. Using a dissecting microscope, the structures of the belowground organs and pruned roots were examined in detail to classify bud types based on their morphology, position, and the characteristics of the attached root system and any remaining aboveground structures (Wang et al., 2018; VanderWeide and Hartnett, 2015). In the present study, tiller, rhizome, and root-derived buds were identified. Bulb buds were not found because the elevation was over 800 m, where bulbous plants are rare (Xia et al., 2004). The quantities of tiller, rhizome, and root-derived buds were recorded for each soil block, and the total quantity of all belowground buds was the sum of these buds in each soil block.

The soil column was rinsed with tap water to identify belowground rhizomes. Belowground rhizomes were separated from their roots and both were placed separately in envelope bags. All shoots harvested in the field and the belowground rhizome and roots were oven-dried at 65°C for 72 h to a constant mass and then weighed.

Soil samples intended for measuring soil bulk weight and soil moisture content were heated to a constant weight at 105°C. The collected soil composites were carefully passed through a 2-mm wire mesh to eliminate vegetative roots, stones, and gravel. Following this, the soil was thoroughly air-dried at room temperature. These air-dried composite samples were then utilized for detailed analysis, including the determination of soil pH, organic carbon content, total nitrogen, total phosphorus, and available nitrogen and phosphorus levels.

Soil total nitrogen and phosphorus were analyzed using an elemental analyzer (VarioEL III; Elementar, Germany) and Mo-Sb colorimetry (UV-2102C; UNICO, Shanghai, China). Soil organic carbon (SOC) was measured via dichromate heating oxidation. Soil available nitrogen (nitrate nitrogen and ammonium nitrogen) was extracted using potassium chloride at a concentration of 2 mol L^−1^ concentration and assessed using the flow injection method (FIAstar 5000 Analyzer, FOSS, DK). Soil available phosphorus was extracted using NaHCO_3_ and measured using Mo-Sb colorimetry (Nelson and Sommers, 1982).

### 2.5 Data analyses

Plant aboveground biomass was the sum of the plant biomass of all species in each subplot. Data from all plots were checked for normality using the Shapiro–Wilk test. If necessary, data were log-transformed to ensure normality.

A generalized linear mixed model was used to analyze the effects of the presence or absence of plateau pikas on plant properties (total belowground bud density, rhizome bud density, root-derived bud density, tiller bud density, aboveground plant biomass, belowground biomass, rhizome biomass, and root biomass), soil physical properties (soil bulk density and soil moisture), and soil chemical properties (soil pH, soil organic carbon, soil total nitrogen, soil total phosphorus, soil available nitrogen, and soil available phosphorus). Belowground buds, plant properties, and soil physical and chemical properties were considered as the response variables, the presence or absence of plateau pikas was a fixed factor, and sampling points were included as random factors.

**Figure 2:**
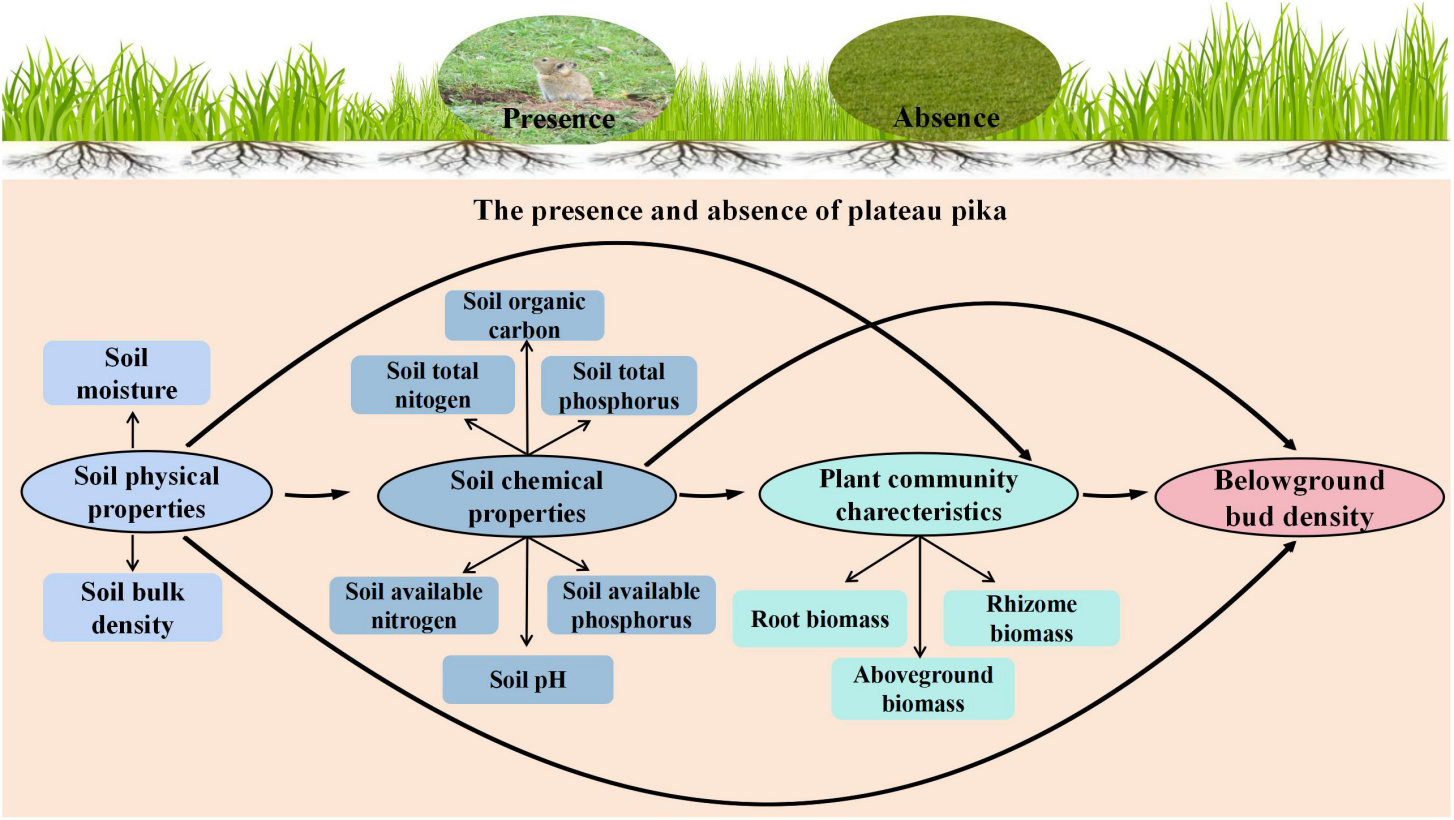
*A priori* structural equation model of soil physicochemical properties and plant biomass variables on belowground bud density in the presence and absence of plateau pika in alpine grasslands.

Pearson’s rank correlation was applied to identify the key factors affecting belowground bud density from the candidate factors (plant properties, soil physical properties, and soil chemical properties). Key factors were used to construct a structural equation model (SEM) in the presence and absence of plateau pikas. A comparison of SEM in the presence and absence of plateau pikas was done to determine whether the presence of plateau pikas changed direct or indirect paths concerning the effects of key factors on plant belowground buds. Based on the knowledge-driven *a priori* model (**Figure 2**), SEM was applied to illustrate the underlying flow of causality between environmental variables and plant belowground bud density.

All statistical analyses were conducted using IBM SPSS 27.0. The figures were plotted using Origin2021. SEM was constructed and analyzed using Amos 17.0.

## 3 Results

### 3.1 Effects of the presence of plateau pika on the total, tiller, rhizome, and root-derived bud densities

**Figure 3:**
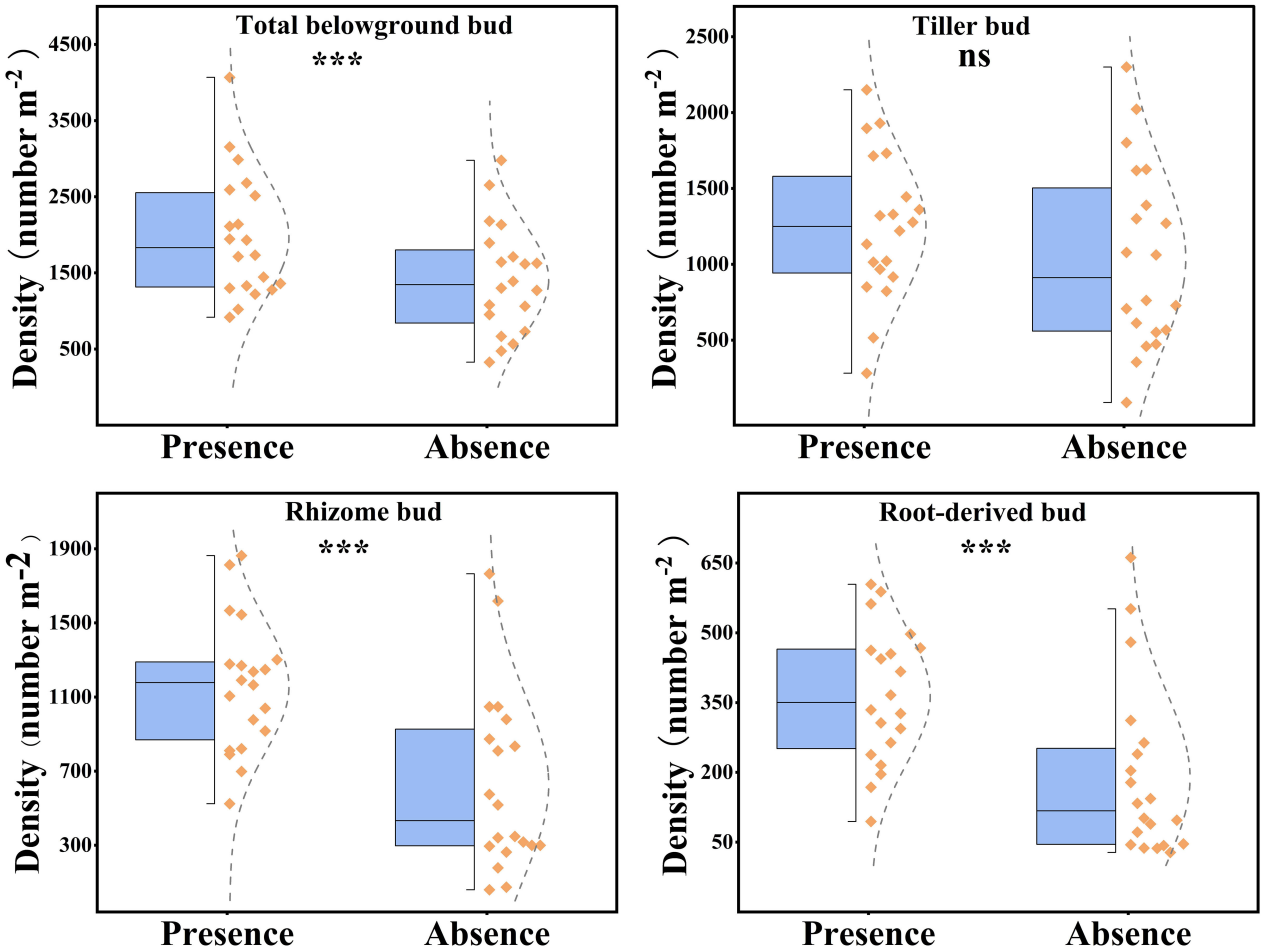
Effect of plateau pika presence on total belowground, rhizome, root-derived, and tiller bud densities in alpine grasslands. Each standard half boxplot displays the maximum, 25% interquartile, and minimum values from top to bottom. Points to the right of the box represent sample data, with those beyond the boxplot limits identified as outliers. Asterisks mark significant differences between the presence and absence of plateau pikas. *p < 0.05; **p < 0.01; ***p < 0.001; ns, *p* > 0.05.

The presence of plateau pikas had a significant effect on the total belowground, rhizome, and root-derived bud densities, whereas it had no significant effect on tiller bud density (**Figure 3**). Total, rhizome, and root-derived bud densities were 49.31%, 84.68%, and 91.73%, respectively, higher in the presence of plateau pikas than in their absence.

**Figure 4:**
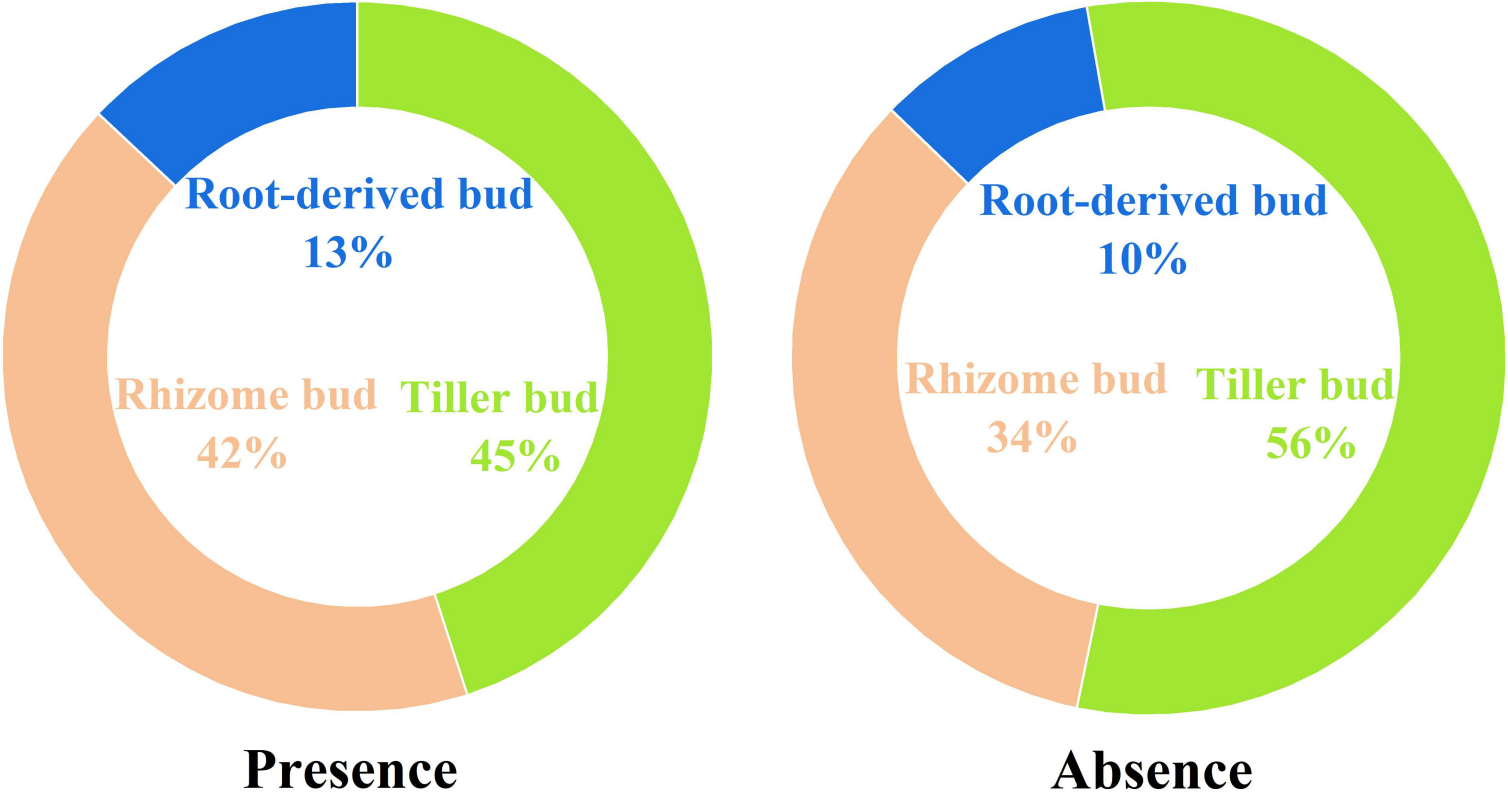
Percentage of tiller, rhizome, and root-derived buds to total belowground buds in the presence and absence of plateau pikas.

The presence of plateau pikas altered the percentage of different bud types relative to the total number of belowground buds (**Figure 4**). The percentage of tiller to total belowground buds decreased from 56.06% in the absence of plateau pikas to 44.83% in their presence, whereas the percentage of rhizome and root-derived buds to total belowground buds increased from 33.79% and 10.15% in the absence of plateau pikas to 41.87% and 13.31% in their presence, respectively.

### 3.2 Effect of the presence of plateau pika on soil physicochemical properties

**Figure 5:**
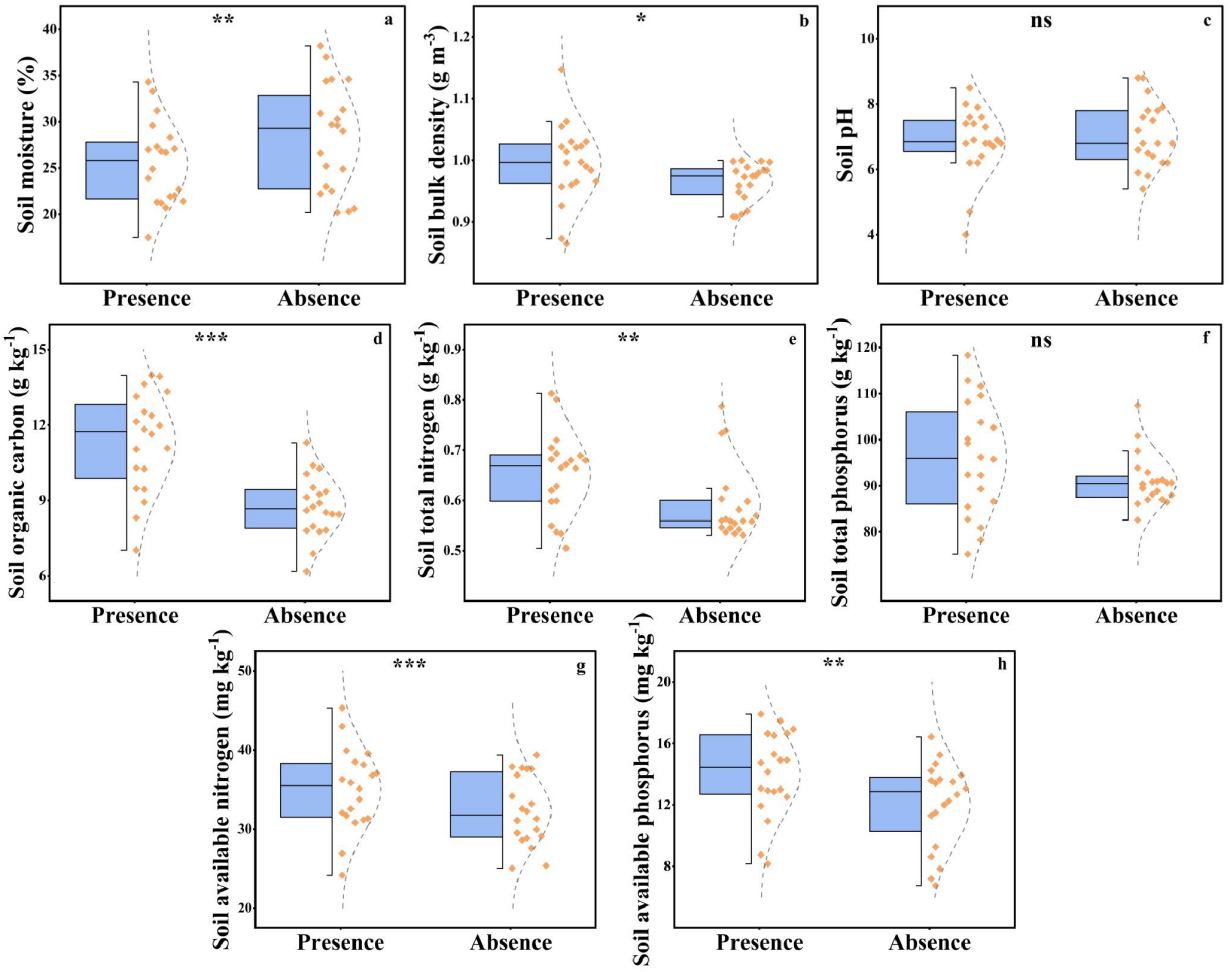
The soil moisture **(a)**, soil bulk density **(b)**, soil pH **(c)**, soil organic carbon **(d)**, soil total nitrogen **(e)**, soil total phosphorus **(f)**, soil available nitrogen **(g)**, and soil available phosphorus **(H)** between the presence absence of plateau pika. From top to bottom of standard half boxplot are the maximum, the third quartile, median, first quartile, and minimum. Asterisks mark significant differences between the presence and absence of plateau pikas. *p < 0.05; **p < 0.01; ***p < 0.001; ns, p > 0.05.

The presence of plateau pikas significantly affected soil moisture, soil bulk density, SOC, soil total phosphorus, soil available nitrogen, and soil available phosphorus but did not significantly affect soil pH and soil total phosphorus (**Figure 5**).

Soil bulk density, SOC, soil total nitrogen, soil available nitrogen, and soil available phosphorus in the presence of plateau pikas were 3.08%, 29.09%, 10.22%, 8.42%, and 16.25% higher than those in the absence of plateau pikas. Soil moisture in the presence of plateau pikas was 9.91% lower than that in the absence of plateau pikas.

### 3.3 Effects of the presence of plateau pika on the species composition of plants with different bud types and plant biomass

The relative frequency of plant species with different bud types showed significant variation between plots with and without plateau pikas (**Supplementary Table S1**). Plants with tiller buds (e.g., *Carex parvula*, *Carex alatauensis*, *Elymus nutans*, and *Poa pratensis*) are highly relative frequencies in plots with and without plateau pikas. In contrast, plants with root-derived buds and rhizome buds are different frequencies between plots with plateau pikas and plot without plateau pikas, in which plants with root-derived buds (e.g., *Taraxacum mongolicum* and *Oxytropis kansuensis*) were significantly higher frequent in plots with plateau pikas than in plots without plateau pikas, whereas plants with some rhizome buds (e.g., *Bistorta vivipara* and *Phlomoides rotata*) are higher frequencies in plots without plateau pikas than in plots with plateau pikas (**Supplementary Table S1**).

**Figure 6:**
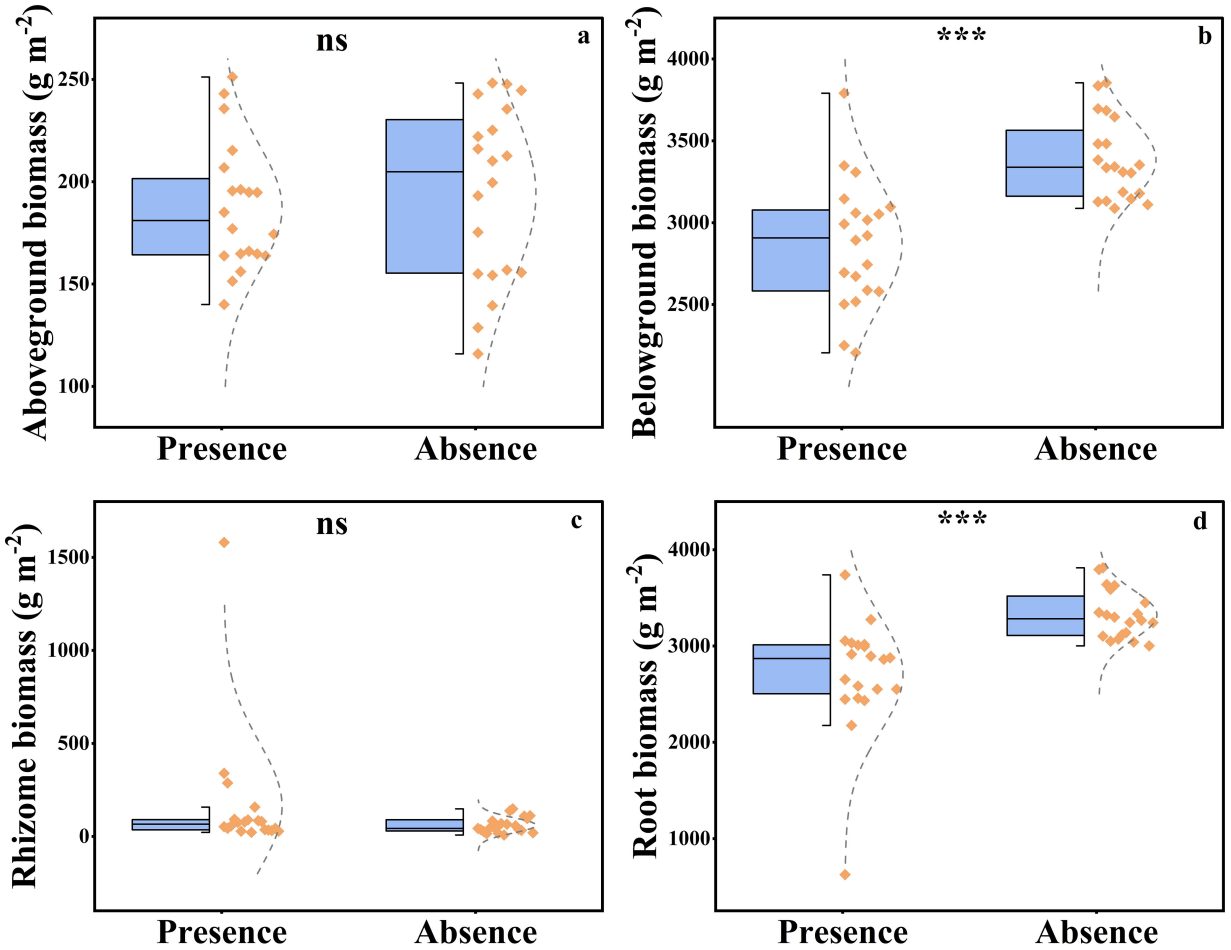
The aboveground biomass **(A)**, belowground biomass **(B)**, rhizome biomass **(C)**, and root biomass **(D)** in the presence and absence of plateau pika. From top to bottom of standard half boxplot are the maximum, the third quartile, median, first quartile, and minimum. Asterisks mark significant differences between the presence and absence of plateau pikas. *p < 0.05; **p < 0.01; ***p < 0.001; ns, p > 0.05.

The presence of plateau pikas significant affected belowground plant biomass and root biomass, whereas it did not affect aboveground plant biomass or rhizome biomass (**Figure 6**). Plant belowground biomass (root biomass and rhizome biomass) and root biomass were 15.22% and 18.57%, respectively, lower in the presence of plateau pikas than in their absence.

### 3.4 Key factors impacting belowground buds in the presence and absence of plateau pikas

**Figure 7:**
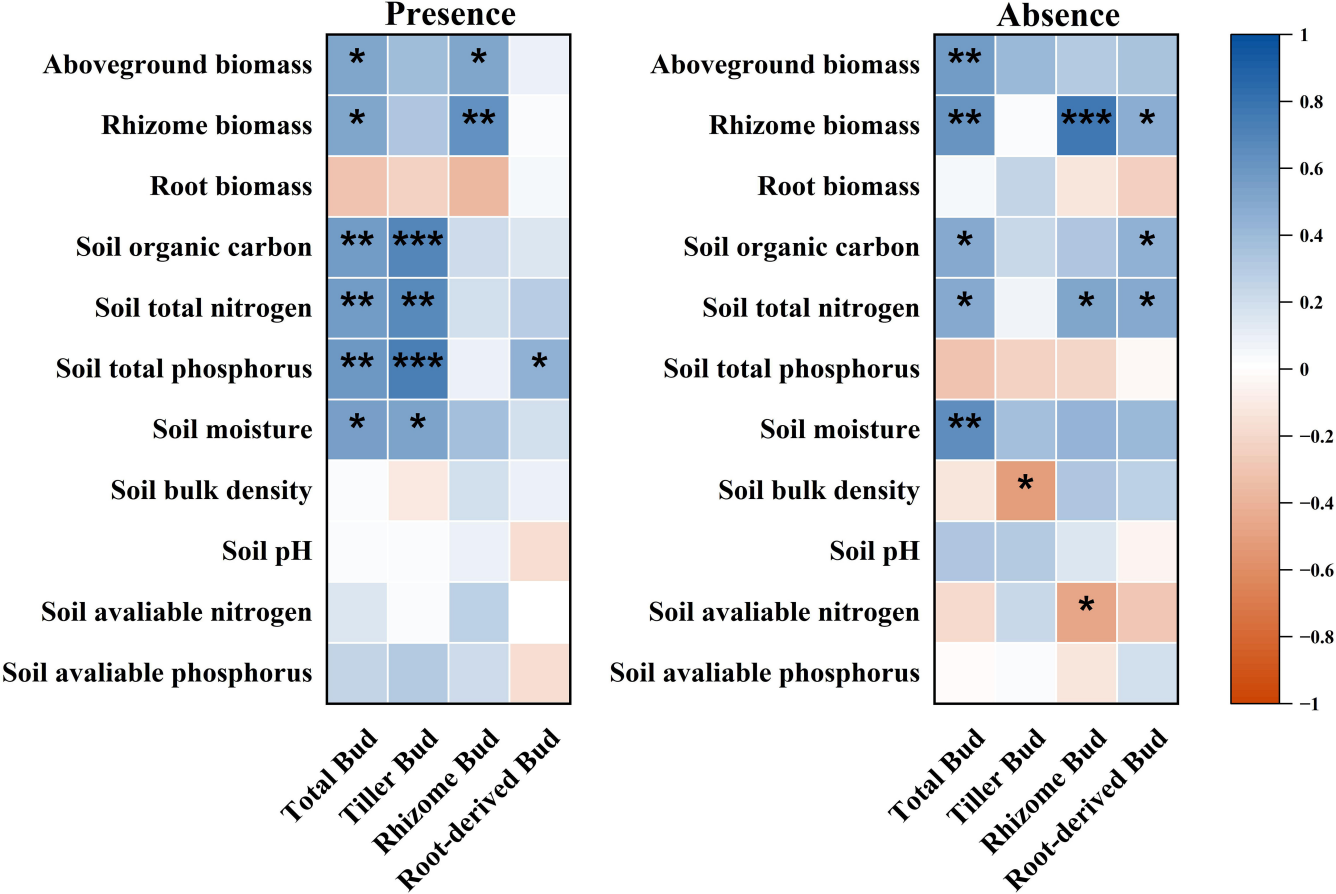
Correlations between belowground bud density and candidate factors (plant community biomass, soil physical, and chemical properties) in the presence or absence of plateau pikas. Asterisks mark significant differences between the presence and absence of plateau pikas. *p < 0.05; **p < 0.01; ***p < 0.001; ns, p > 0.05.

Total belowground bud density was significantly correlated with plant aboveground biomass, rhizome biomass, SOC, soil total nitrogen, soil total phosphorus, and soil moisture in the presence of plateau pikas. In contrast, it was significantly correlated with rhizome biomass, SOC, and soil moisture in the absence of plateau pikas (**Figure 7**). Total belowground bud density was not correlated with plant belowground biomass, root biomass, soil bulk density, soil pH, soil available nitrogen, and soil available phosphorus in the presence or with aboveground biomass, belowground biomass, root biomass, soil total nitrogen, soil total phosphorus, soil bulk density, soil pH, soil available nitrogen, and soil available phosphorus in the absence of plateau pikas (**Figure 7**).

Tiller bud density was significantly correlated with SOC, total nitrogen, total phosphorus, and moisture in the presence of plateau pikas and was only significantly correlated with soil bulk density in the absence of plateau pikas. Rhizome bud density was significantly correlated with plant aboveground and rhizome biomass in the presence and with rhizome biomass, soil total nitrogen, and soil available nitrogen in the absence of plateau pikas. Root-derived bud density was significantly correlated with soil total phosphorus in the presence and with rhizome biomass, SOC, and soil total nitrogen in the absence of plateau pikas.

### 3.5 Pathways impacting belowground bud density in the presence and absence of plateau pikas

Tiller and root-derived bud densities were key factors in the absence and presence of plateau pikas, respectively, and rhizome bud density had two key factors in the presence of plateau pikas, which did not meet the basic conditions of the third-order path to construct SEM, indicating that SEM was not simultaneously constructed to compare the effects of plant, soil physical, and soil chemical pathway properties on tiller, root-derived, and rhizome bud densities.

**Figure 8:**
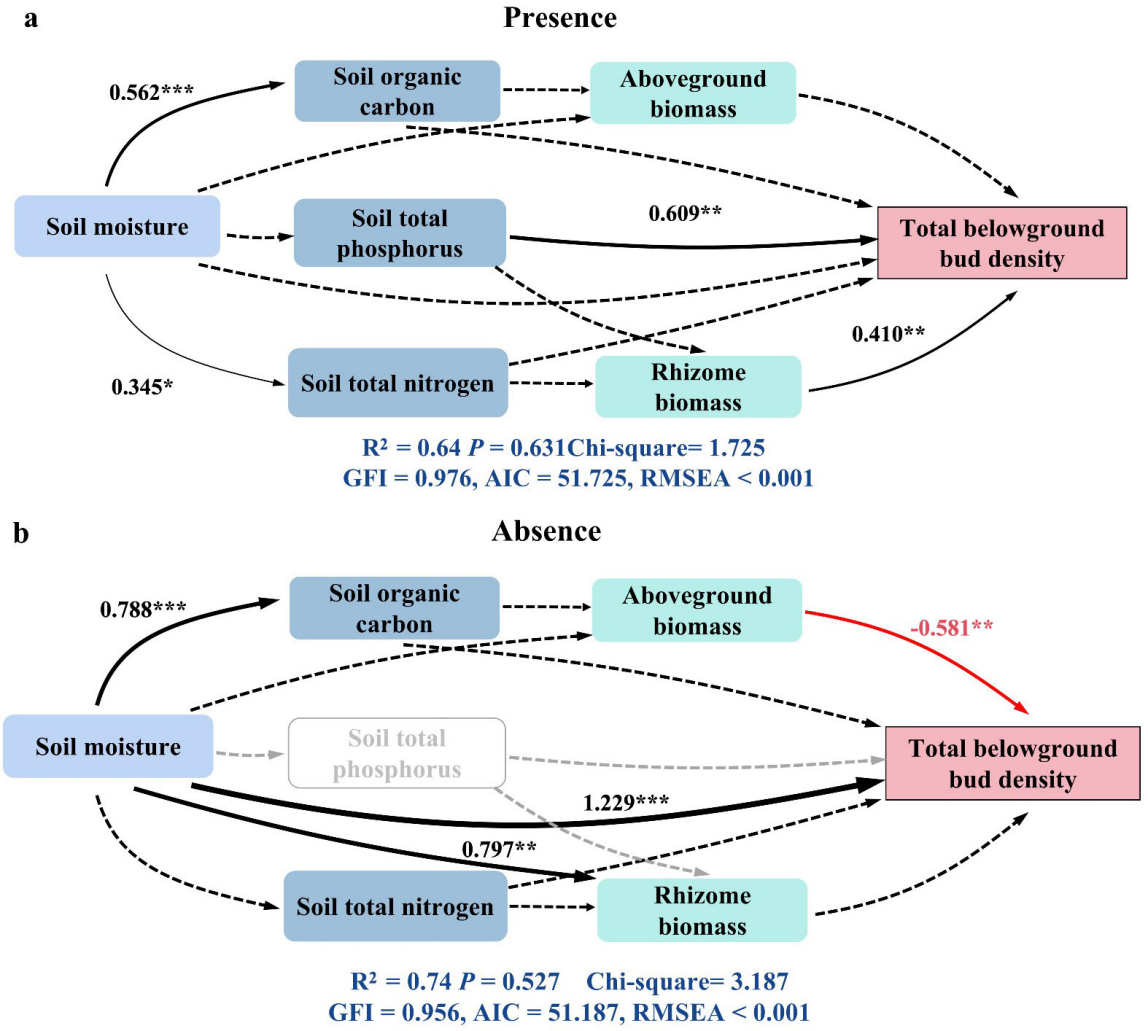
Structural equation modeling (SEM) was used to examine the direct and indirect effects of soil physical properties (soil moisture), soil chemical properties (soil organic carbon, total phosphorus, and total nitrogen), and plant biomass (aboveground and rhizome biomass) on total belowground bud density in the presence **(a)** and absence **(b)** of plateau pikas. Positive and negative paths are represented by blank and red arrows, respectively, while dotted arrows indicate non-significant effects. Arrow width corresponds to the standardized regression coefficients and SEM coefficients. Gray-dotted arrows denote nonexistent pathways. The proportion of variance explained (*R* ^2^) for each response variable is included in the models. For clarity, only significant paths (*p* < 0.05) are displayed. Asterisks mark significant differences between the presence and absence of plateau pikas. *p < 0.05; **p < 0.01; ***p < 0.001; ns, p > 0.05.

SEM could be used to compare the effects of plant properties, soil physical properties, and soil chemical properties on the total plant belowground bud density in the presence or absence of plateau pikas. The SEM results explained 64% and 74% (*R* ^2^ = 0.64 and 0.74) of the variance in total belowground bud density in the presence and absence of plateau pikas, respectively. In the presence of plateau pikas, soil total phosphorus and rhizome biomass had direct positive effects on total belowground bud density. In the absence of plateau pikas, soil moisture had direct positive effects on total belowground bud density, while the aboveground biomass had direct negative effects on total belowground bud density. These results demonstrated that the presence of plateau pikas altered the relationship between total belowground bud density and key factors, in which soil moisture and aboveground plant biomass were important for alpine grasslands to regenerate plant populations and maintain plant community components in the absence of plateau pikas. Furthermore, soil total phosphorus and rhizome biomass were key to regenerating plant populations and maintaining plant community components in the absence of plateau pikas (**Figure 8**).

## 4 Discussion

Small semi-fossorial herbivores are important drivers of alpine grassland succession (Davidson et al., 2012). Thus, this study used the plateau pika as a focal animal to investigate the impact of small semi-fossorial herbivores on different types of belowground bud density and total belowground bud density. The aim is to provide insights that support plant population regeneration, community stability, and positive succession in alpine grasslands when plateau pikas are present.

This study showed that plateau pikas’ presence resulted in higher rhizome and root-derived bud densities and had no effect on tiller bud density in alpine meadows, which disagreed with the first hypothesis and the responses of rhizome, tiller, and root-derived bud densities to large-herbivore grazing with lower rhizome, tiller, and root-derived bud densities in grasslands (Zhao et al., 2017). These differences are ascribed to bud position in the soil profile and the response of plant aboveground biomass to herbivore size. Tiller buds are generally located in shallower soil layers than rhizome and root-derived buds (Klimešová et al., 2018; Ott et al., 2019) and are more easily induced to become new shoots than rhizome and root-derived buds in response to plant compensatory growth, which results in a decrease in tiller bud density. Herbivore consumption can stimulate plants to produce more tiller buds on one shoot to recruit the aboveground population of clonal fragments (Wang et al., 2020). In addition, plateau pikas are lighter than sheep; the trampling of plateau pikas does not damage the tiller bud and does not affect soil compactness, whereas the trampling of sheep usually damages tiller buds and increases soil compactness (Zhao et al., 2017), and higher soil compactness is less conducive to producing new tiller buds (Heggenes et al., 2017). Thus, the results of the statistical analysis indicated that the presence of plateau pikas appears to have a zero net effect on tiller bud density, which stems from the fact that the decrease in tiller buds associated with plant compensatory growth and trampling damage is offset by the increase in tiller buds as a consequence of plateau pikas stimulation.

Changes in rhizome and root-derived bud densities are related to changes in aboveground plant biomass in the presence of herbivores. The presence of plateau pikas has been verified to have no influence on plant aboveground biomass when paired plots were applied to samples of the same alpine meadow type on a small scale (Pang and Guo, 2017; Pang et al., 2020; Wang et al., 2022), whereas large-herbivore grazing decreased plant aboveground biomass. Thus, in response to plant compensatory growth, more belowground buds are required in the presence of large herbivores than in the presence of plateau pikas (Heggenes et al., 2017). The tiller bud is sufficient to respond to plant compensatory growth to maintain plant aboveground biomass in the presence of plateau pikas because their presence has little effect on plant aboveground biomass. Thus, the rhizome and root-derived bud in the presence of plateau pikas are not necessary to respond to plant compensatory growth, inversely, and the plant compensatory growth encourage plants to produce more rhizome and root-derived buds (Wang et al., 2022), leading to an increase in rhizome and root-derived bud densities. In addition to tiller buds, some rhizome and root-derived buds in the presence of large herbivores need to form new shoots to respond to plant compensatory growth because of large-herbivore grazing in relation to lower plant aboveground biomass (Grasses, 1989; Zhao et al., 2017), resulting in a decrease in rhizome and root-derived bud densities when large herbivores are present.

Higher rhizome and root-derived bud densities led to a higher total belowground bud density in the presence of plateau pikas. We also found that the presence of plateau pikas leads to a higher percentage of rhizome and root-derived buds to total belowground buds and a lower percentage of tiller buds to total belowground buds, which disagreed with the second hypothesis. These results demonstrated that the presence of plateau pikas increases the contribution of rhizome and root-derived buds to the total belowground buds, whereas it decreases the contribution of tiller buds to the total belowground buds. In this case, the increased contribution of rhizome and root-derived buds in the presence of plateau pikas can be attributed to the two mechanisms. First, plateau pika disturbance can increase soil nutrient availability and facilitate the rhizome elongation of perennial rhizomatous plants (Wang et al., 2020), which provide more changes for rhizome to produce more buds (Pan et al., 2004; Fu and Shen, 2017). Second, the higher annual non-graminoid forbs caused by plateau pika disturbance (Wang et al., 2018) may contribute to the seasonally formation of root-derived buds (Hartnett et al., 2006). These indicate that the presence of plateau pikas at a density of 550 to 650 per ha is advantageous for the regeneration of plant populations and the maintenance of plant communities in alpine grasslands.

We further found that plateau pikas altered the relationship between total belowground bud density and environmental variables (soil moisture and total phosphorus), which agreed with the third hypothesis. In the presence of plateau pikas, soil total phosphorus had direct positive effects on total belowground bud density. In the absence of plateau pikas, soil moisture had a direct positive effect and plant aboveground biomass had a direct negative effect on total belowground bud density. Alpine grasslands generally have a root mat (consisting of compact plant roots) in the topsoil horizon, which impedes water infiltration (Pang et al., 2020). However, plateau pikas’ burrowing activity disrupts root mats, enhancing water infiltration (Wilson and Smith, 2015). Soil moisture was insufficient to develop more buds in the absence of plateau pikas because the greater the soil moisture, the more belowground buds were produced (Ma et al., 2023). In contrast, it was sufficient to develop more buds in the presence of plateau pikas because of higher rainfall infiltration. Thus, soil moisture directly affected total belowground bud density in the absence of plateau pikas and had no effect on total belowground bud density in the presence of plateau pikas. Some phosphorus in alpine grasslands leaches from top to deep soils beyond the plant-root zone (Pang et al., 2021; Xiao et al., 2022), which causes soil total phosphorus to have a direct effect on the total belowground bud density in the presence of plateau pikas, although it has no direct effect in the absence of plateau pikas. These results suggested that phosphorus supplementation facilitates higher total plant belowground bud density and improves plant population regeneration and plant community maintenance, which is helpful for alpine grassland sustainability when plateau pikas are present.

The findings of this study show that the presence of plateau pikas leads to higher rhizome and root-derived bud densities, facilitating the plant population regeneration and maintaining the stability of plant communities in alpine grasslands of the Qinghai–Tibetan Plateau, and this is also reported in the presence of Tatra marmot (Ballova et al., 2019), whereas it is not agreement with the presence of North American Plains pocket gophers (Reichman and Seabloom, 2002) and plateau zokors (Hu et al., 2017). These suggest that the effect of semi-fossorial herbivores on the plant regeneration varies with grassland types in various environments and disturbance behavior of small herbivores. Although this study uses the observed data to analyze the relationship between phosphorus and belowground bud density, more studies are needed to verify the causal link between phosphorus and belowground bud density through manipulative experiments in the future.

## 5 Conclusions

We used paired plots across various habitats to investigate the effect of the presence of plateau pikas on different types of belowground bud density and total belowground bud density. The results of this study revealed that the presence of plateau pikas contributes to an increase in the densities of rhizome and root-derived buds. However, their presence does not result in any significant overall change in the density of tiller buds. Higher rhizome and root-derived buds led to an increase in total belowground bud density in the presence of plateau pikas. The presence of plateau pikas changed the relationship between environmental factors and total belowground bud density, in which soil phosphorus had no direct effect on total belowground bud density in the absence of plateau pikas but direct positive effect on in the presence of plateau pikas, indicating that phosphorus supplementation may be a potential management strategy to enhance the regenerative capacity of plant populations in plateau pika ecosystems. This study suggests that plateau pikas are beneficial to the plant population regeneration and maintaining the stability of plant communities in alpine grasslands of the Qinghai–Tibetan plateau, indicating that the managing plateau pikas should focus on controlling their densities, rather than eradication.

## Data Availability

The raw data supporting the conclusions of this article will be made available by the authors, without undue reservation.
