# Supplementary material for: Plateau pikas lead to higher rhizome and root-derived bud densities and their contribution to total belowground bud density in alpine grasslands
Source: Front Plant Sci. 2025 May 20;16:1567822. doi: 10.3389/fpls.2025.1567822 (PMC12130033; doi:10.3389/fpls.2025.1567822)
Supplement: Supplementary file 1 [file Table1.docx]

**Supplementary Table 1: Relative frequency of plant species in plots with and without plateau pikas.**

| **Species name** | **Bud Type** | **With plateau pikas** | **Without plateau pikas** | **Relative frequency (%)** | |
| --- | --- | --- | --- | --- | --- |
|  |  | **n/20** | **n/20** | **With** | **Without** |
| *Carex parvula* | Tiller bud ＋ Rhizome bud | 19 | 17 | 95% | 85% |
| *Carex alatauensis* | Tiller bud ＋ Rhizome bud | 19 | 15 | 95% | 75% |
| *Anemone rivularis* | Rhizome bud | 18 | 8 | 90% | 40% |
| *Gentiana straminea* | Tiller bud | 7 | 6 | 35% | 30% |
| *Potentilla fragarioides* | Rhizome bud | 12 | 8 | 60% | 40% |
| *Elymus nutans* | Tiller bud | 18 | 12 | 90% | 60% |
| *Anaphalis lactea* | Rhizome bud | 16 | 9 | 80% | 45% |
| *Saussurea superba* | Rhizome bud | 16 | 11 | 80% | 55% |
| *Anemone trullifolia var.* | Rhizome bud | 14 | 7 | 70% | 35% |
| *Poa pratensis* | Tiller bud ＋ Rhizome bud | 18 | 10 | 90% | 50% |
| *Stipa purpurea* | Tiller bud | 10 | 11 | 50% | 55% |
| *Melissitus ruthenicus* | Rhizome bud ＋ Root-derived bud | 16 | 8 | 80% | 40% |
| *Oxytropis kansuensis* | Root-derived bud | 17 | 5 | 85% | 25% |
| *Oxytropis ochrocephala* | Root-derived bud | 15 | 10 | 75% | 50% |
| *Bistorta vivipara* | Rhizome bud | 8 | 15 | 40% | 75% |
| *Knorringia sibirica* | Rhizome bud | 15 | 7 | 75% | 35% |
| *Potentilla multifida* | Rhizome bud | 12 | 6 | 60% | 30% |
| *Potentilla angustiloba* | Rhizome bud | 13 | 7 | 65% | 35% |
| *Potentilla bifurca* | Rhizome bud | 11 | 5 | 55% | 25% |
| *Ranunculus brotherusii* | Root-derived bud | 14 | 4 | 70% | 20% |
| *Phlomoides rotata* | Rhizome bud | 9 | 12 | 45% | 60% |
| *Taraxacum mongolicum* | Root-derived bud | 19 | 3 | 95% | 15% |

**Continuation of Supplementary Table 1: Relative frequency of plant species in plots with and without plateau pikas.**

| **Species name** | **Bud Type** | **With plateau pikas** | **Without plateau pikas** | Relative frequency (%) | |
| --- | --- | --- | --- | --- | --- |
|  |  | **n/20** | **n/20** | **With** | **Without** |
| *Aster flaccidus* | Rhizome bud | 15 | 5 | 75% | 25% |
| *Cirsium souliei* | Root-derived bud | 15 | 18 | 75% | 90% |
| *Leontopodium brachyactis* | Tiller bud ＋ Rhizome bud | 12 | 9 | 60% | 45% |
| *Saussurea hieracioides* | Tiller bud ＋ Rhizome bud | 14 | 6 | 70% | 30% |
| *Saussurea pulchra* | Tiller bud ＋ Rhizome bud | 16 | 8 | 80% | 40% |
| *Taraxacum mongolicum* | Root-derived bud | 19 | 2 | 95% | 10% |
| *Carex humilis* | Tiller bud ＋ Rhizome bud | 10 | 9 | 50% | 45% |
| *Carex moorcroftii* | Tiller bud ＋ Rhizome bud | 13 | 7 | 65% | 35% |
| *Carex capillifolia* | Tiller bud ＋ Rhizome bud | 15 | 6 | 75% | 30% |
| *Euphorbia micractina* | Rhizome bud | 18 | 5 | 90% | 25% |
| *Gentiana macrophylla* | Rhizome bud | 15 | 10 | 75% | 50% |
| *Gentiana scabra* | Rhizome bud | 14 | 6 | 70% | 30% |
| *Leymus secalinus* | Tiller bud | 9 | 9 | 45% | 45% |
| *Tibetia himalaica* | Tiller bud | 10 | 9 | 50% | 45% |
| *Thalictrum aquilegiifolium* | Rhizome bud | 16 | 7 | 80% | 35% |
| *Plantago asiatica* | Root-derived bud | 15 | 6 | 75% | 30% |

“n” represents the number of times each plant species appears across the 20 plots with plateau pikas and 20 plots without plateau pikas.

Relative frequency (%): Percentage of plots with plant species occurrence.
